# Cloning, expression, purification and characterization of lipase from *Bacillus licheniformis*, isolated from hot spring of Himachal Pradesh, India

**DOI:** 10.1007/s13205-016-0369-y

**Published:** 2016-02-08

**Authors:** Gagandeep Kaur, Amninder Singh, Rohit Sharma, Vinay Sharma, Swati Verma, Pushpender K. Sharma

**Affiliations:** 1 Department of Biotechnology, Sri Guru Granth Sahib World University, Fatehgarh Sahib, Punjab India; 2 NABI, Mohali, Punjab India; 3 Department of Microbiology, Maharaja Ganga Singh University, Bikaner, Rajasthan India

**Keywords:** Lipase, *Bacillus licheniformis*, Metal ions, Detergents, Inhibitors, Solvents

## Abstract

In the present investigation, a gene encoding extracellular lipase was cloned from a *Bacillus licheniformis*. The recombinant protein containing His-tag was expressed as inclusion bodies in *Esherichia coli* BL21DE3 cells, using pET-23a as expression vector. Expressed protein purified from the inclusion bodies demonstrated ~22 kDa protein band on 12 % SDS-PAGE. It exhibited specific activity of 0.49 U mg^−1^ and % yield of 8.58. Interestingly, the lipase displayed activity at wide range of pH and temperature, i.e., 9.0–14.0 pH and 30–80 °C, respectively. It further demonstrated ~100 % enzyme activity in presence of various organic solvents. Enzyme activity was strongly inhibited in the presence of β-ME. Additionally, the serine and histidine modifiers also inhibited the enzyme activities strongly at all concentrations that suggest their role in the catalytic center. Enzyme could retain its activity in presence of various detergents (Triton X-100, Tween 20, Tween 40, SDS). Sequence and structural analysis employing *in silico* tools revealed that the lipase contained two highly conserved sequences consisting of ITITGCGNDL and NLYNP, arranged as parallel β-sheet in the core of the 3D structure. The function of these conserve sequences have not fully understood.

## Introduction

Lipases are triacylglycerol hydrolases (E.C. 3.1.1.3) that catalyzes variety of conversion reactions, ranging from interesterification, esterification, alcoholysis, acidolysis and aminolysis (Gupta et al. 2004; Hayes 2004; Kim et al. 2002; Nagao et al. 2001; Saxena et al. 2003). They are frequently used in various industries including detergents, leather, dairy, baking and pharmaceutical (Jaeger and Eggert 2002). Thermostable lipases have received much more attention, as they remain stable at high temperature. Reactions carried out at higher temperature lead to higher diffusion rate, increases solubility of lipids and other hydrophobic substrates in water, can reduce the risk of contamination (Annamalai et al. 2011). They display broader range of substrate specificity, tolerance towards extreme of acidic and alkaline conditions, and solvents (Gupta et al. 2004; Haki and Rakshit 2003). The most remarkable sources of thermostable lipases are bacteria (Jaeger and Eggert 2002). Importantly, the *Bacillus* sp. is known to produce commercially important lipases (Chakraborty and Raj 2008). Recently, Shariff et al. (2011) characterized a lipase from *Bacillus* sp. L2 that showed lipase activity at wide range of pH and temperature. In another study, a lipase was characterized from *Bacillus* *licheniformis* strain that was isolated from the mangrove sediments. Interestingly, this enzyme showed stability up to 70 °C and pH 12.0 (Annamalai et al. 2011). More recently, Gohel et al. (2013) found better stability and activity of thermophilic lipases in its immobilized form. In general, a number of lipases have been characterized from various bacterial species and cannot be discussed here.

In the present investigation, we report cloning, expression, purification and characterization of lipase from bacteria, and interestingly we observed that the present lipase exhibits facet of industrial relevance.

## Materials and methods

### Screening of lipase-producing bacterial strain

Soil sample was collected from hot springs of Manikaran (average water temperature: 60–65 °C), Himachal Pradesh, India (32°1′40.34″N and 77°2′52.95″E) in sterile oakridge centrifuge tube. The soil sample was dried in an oven maintained at 50 °C for screening thermostable lipase-producing strain. One gram soil was dissolved in water and diluted appropriately to 10^−9^ dilutions, followed by spreading on LB (Luria broth) agar plates, containing 1 % emulsified tributyrin. The plates were incubated at 40 °C for 2 days. The colony displaying hydrolytic zone on substrate plate was streaked further for selecting single colony.

### DNA extraction and 16S rRNA amplification

The colony displaying zone of hydrolysis in LB agar tributyrin plates was cultured overnight in 5 ml LB at 37 °C in shaker (220 rpm). Genomic DNA was extracted from overnight grown culture employing standard phenol–chloroform method. Purified DNA was used for PCR amplification of the 16S rRNA gene, using universal primers: 27F (5′-AGAGTTTGATCMTGGCTCAG-3′) and 1642R (5′-CGGYTACCTTGTTACGAC-3′), respectively. The following thermal cycling conditions were used to amplify the 16S rRNA gene, 94 °C for 4 min, followed by 30 cycles of 94 °C for 50 s, 55 °C for 50 s, 72 °C for 2 min, with final extension of 7 min at 72 °C. The 25 µl PCR mix contained 1 µl (0.1 µg) DNA, 1 μl each for ward and reverse primers (2.5 µM), 200–400 µM dNTPs mix (Thermo Scientific), 1U *Taq* DNA polymerase (Thermo Scientific) and 1× reaction buffer (Thermo Scientific). PCR-amplified product was checked on a 1.5 % agarose gel, and purified from gel employing XcelGen DNA Gel/PCR Purification kit (Xcelris genomics). The amplified product was cloned in pGEM-T easy vector (Promega, USA) as per manufacturer’s instruction. The recombinant plasmid DNA was extracted from white colonies employing Hi Yield™ Plasmid DNA Mini Kit (Real Genomics) and sequenced using M13 forward and reverses primer.

### PCR amplification and cloning of the lipase gene

The analysis of the 16 SrRNA gene sequence revealed that the strain producing lipase was *Bacillus licheniformis*. After careful analysis of the *Bacillus licheniformis* genome at NCBI, specific forward and reverse primers were designed to amplify the corresponding lipase gene, F:5′ATTCAGCATATGGAAGAGGATTTGAACGAA3′R:5′ATTCAGCTCGAGGCCCTCCAGCCGCCCGTA3′ containing restriction sites Nde1 and Xho1 in forward and reverse primers, respectively. The lipase gene (**lip P1**) was amplified employing following reaction conditions, initial denaturation at 94 °C for 4 min, followed by subsequent 35 cycles of 94 °C for 50 s, 50 °C for 1 min, 72 °C for 1 min and final extension at 72 °C for 7 min. The amplified gene product was checked on 1.5 % agarose gel and extracted by gel extraction kit. The eluted DNA fragment was ligated into pGEM-T-easy vector, as per manufacturer instructions and transformed into *E. coli* competent cells. The white colonies (recombinant clones) carrying a recombinant plasmid were cultured overnight in 5 ml LB having ampicillin (100 μg ml^−1^) followed by plasmid DNA extraction and sequencing of lipase gene using M13 forward and reverse primers.

### Protein expression and purification

The **lip P1** gene without the terminal signal was further sub-cloned in frame into the pET-23a expression vector, after digesting the amplified DNA fragment and pET 23a plasmid with Nde1 and Xho1. The two DNA fragments were ligated and transformed into the *E. coli* BL-21 DE3 cells. The recombinant clones were selected on LB agar plates having ampicillin (100 µg/ml) and chloramphenicol (25 µg/ml). Transformed colonies were further selected randomly for the presence of insert by colony PCR methods. The positive colony was cultured overnight initially into 5 ml LB medium, having appropriate antibiotics. Next day 1 % overnight grown culture was inoculated into 500 ml LB containing appropriate antibiotics. The culture was induced by addition of 0.005 M IPTG when OD_600nm_ reached ~0.4. The cells were pelleted down, lysed and centrifuged. The SDS-PAGE was performed (sodium dodecyl sulfate–polyacrylamide gel electrophoresis) with both the pellet and the soluble fractions, to know the localization of the recombinant protein. The expressed protein was recovered from inclusion bodies by dissolving it in 8 M urea, and the denatured protein was refolded by dialyzing it in gradient buffer consisting of 100, 50 and 10 mM sodium phosphate. The clear supernatant obtained after dissolving the inclusion bodies was passed through the equilibrated Ni-NTA column, and the eluted protein was collected in fractions of 1.5 ml in 2-ml centrifuge tubes, and checked on 12 % SDS-PAGE. The fractions showing similar pattern of protein bands in the gel were pooled and dialyzed. The protein concentration was estimated by Bicinchoninic acid method (BCA) kit (Banglore Genei, India) and Bovine serum albumin (BSA) was used as standard.

#### **Note**

All steps for the protein purifications were carried out at 4 °C.

### Enzyme assay

The enzyme was assayed by modified method of Sigurgisladottir et al. (1993). The final reaction mixture (1 ml) contained 0.8 ml of 0.05 M sodium phosphate buffer (pH 8.0), 0.1 ml enzyme of appropriate dilution, and 0.1 ml of 0.001 M *p*-nitrophenyl laurate. The reaction was carried out at 60 °C for 1 h, the enzyme reaction was de-activated by addition of 0.1 M Na_2_CO_3_ (0.25 ml), followed by centrifugation. The enzymatic activity was recorded at 420 nm in UV/Vis spectrophotometer (JENWAY 6505 UK). One unit of enzyme activity was defined as the amount of enzyme which liberates 1 µmol of *p*-nitrophenol from pNP-laurate as substrate per min under standard assay conditions. The total enzyme activity was expressed in Unit/ml and specific activity was expressed as Unit/mg of protein.

### Biochemical characterization

#### Effect of temperature and pH on lipase activity and stability

To determine the optimum temperature, purified lipase was assayed at various temperatures (30–80 °C). The enzyme stability was studied by incubating the purified enzyme at 60 °C. The enzyme without incubation was taken as control (100 %). The lipase activity was measured at intervals of 20, 40, 60 and 120 min. Reaction mix without enzyme served as a blank. Next, the pH optimum was determined by assaying the purified lipase in presence of a range of pH (6.0–14.0) at 60 °C.

#### Effect of additives and chemical modifiers on lipase activity

The effect of various metal ions (1 and 10 mM each), detergents (1, 5, and 10 % W/V each), enzyme inhibitors (1, 5, 10, and 20 mM each) and organic solvents (1, 5, and 10 % V/V each) were studied by adding these modifiers and additives separately into the reaction mix. The enzyme assay was performed according to standard assay conditions, and carried out in both the presence and absence of chemical modifiers. The reaction mix with respective additives/chemical modifiers but without enzyme served as blank. The sample without any additive/modifier was taken as control (100 %).

#### Nucleotide sequencing and gene submission

Both 16S rRNA and lipase gene were sequenced using universal M13 forward and reverse primers. The nucleotide sequencing was carried out by Bangalore Genei, India using an automated AB1 3100 genetic analyzer that uses fluorescent label dye terminator, based on dideoxy chain termination method. The 16S rRNA gene was submitted to NCBI under the accession number KM438034, and the lipase nucleotide sequence submitted was assigned gene accession number KF815062.

### *In silico* characterization

To gain structural insights, homology studies were carried out. Using ConSeq server (http://consurf.tau.ac.il/), the homologues were collected from the UNIREF90, and best hits were considered for further studies. Multiple Sequence Alignment (MSA) was performed using MAFFT, using default values of ConSeq server. Conservation scores were calculated by the Bayesian method. Further the secondary and tertiary structure analysis was carried out in PSIPRED (http://bioinf.cs.ucl.ac.uk/psipred/) and Swiss Model (http://swissmodel.expasy.org) server respectively. The target lipase sequence displayed poor sequence similarity with most of the earlier reported lipases for which X-ray structures were available, and could be modeled using template PDB ID: 3BZW of a putative lipase reported from *Bacteroides thetaiotaomicron* that showed ~20 % homology with the lipase under investigation.

## Results

### Screening and identification of the lipase-producing strain

**Fig. 1:**
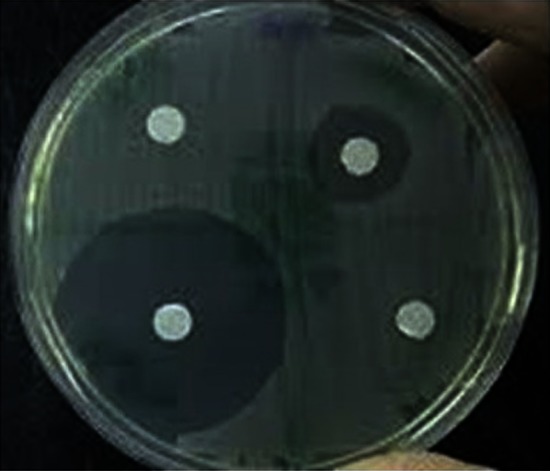
Isolated strain demonstrating the extracellular lipase activity in 1 % emulsified tributyrin plate

Screening of hot spring soil for the lipase-producing strain resulted in isolation of bacterial strain that showed a clear zone of hydrolysis around the colonies (Fig. 1). Genomic DNA extracted showed an intact band (>23 kb) on 0.8 % agarose gel (data not shown). Further, the 16S rRNA gene amplified from the genomic DNA showed amplified band corresponding to ~1.5 kb. BLAST N analysis of the 16S rNRA nucleotide revealed >95 % homology to the 16S rRNA sequences of the *Bacillus licheniformis*.

### Cloning, sequencing, analysis and expression studies

**Fig. 2:**
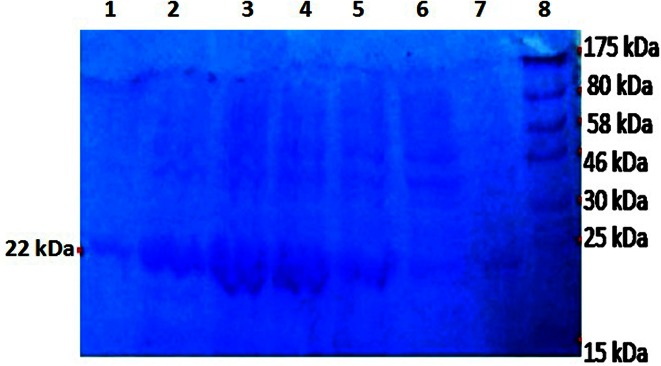
SDS-PAGE gel stained with Coomassie brilliant blue showing *B. licheniformis* lipase. *Lane 1* Purified lipase, *lanes 2–7* expression of lipase gene induced with IPTG, *lane M* Protein molecular weight marker

Lipase gene Lip P1 corresponding to a size of ~0.6 kb was amplified cloned and sequenced. The sequencing of the lipase gene confirmed an ORF (open reading frame) consisting of 0.636 kb that encodes an extracellular lipase of 211 amino acids long. BlastN at NCBI demonstrated that it had shared ~99 % identity with *B. licheniformis* ATCC 14580 (NCBI accession number: CP000002.3), *B. licheniformis* DSM 13 (AE017333.1), *B. licheniformis* Pb-WC11006 (JX164087.1), *B. licheniformis* Pb-WC09005 (HM006905.1), *B. licheniformis* N22 (GU086425.1). Further analysis of the gene using Signal P 4.1 server (Petersen et al. 2011) revealed a terminal signal sequence of 30 amino acids, and hence the mature lipase consists of 181 amino acids. The lipase gene Lip P1 without terminal signal expressed in *E. coli* BL21 (DE3) showed an induced protein band at ~22 kDa, as judged by Coomassie brilliant blue staining of SDS-PAGE (Fig. 2). The purification profile of the protein obtained through the Ni–NTA affinity chromatography is presented in Table 1. From purification table it becomes evident that protein had a specific activity of 0.49 U mg^−1^, and had yield of 8.5 %.

**Table 1:** Summary of the purification profile of lipase from *B. licheniformis*

Steps	Volume (ml)	Total protein (mg)	Total activity (U/ml)	Specific activity (U mg^−1^)	Purification (fold)	Yield (%)
Homogenate	40	9.48	2.75	0.29	1	100
Dialysed	35	8.29	2.72	0.32	1.1	98.9
Purified protein	2	0.47	0.23	0.49	1.7	8.5

### Biochemical characterization of the purified lipase

#### Effect of temperature and pH on lipase activity and stability

**Fig. 3:**
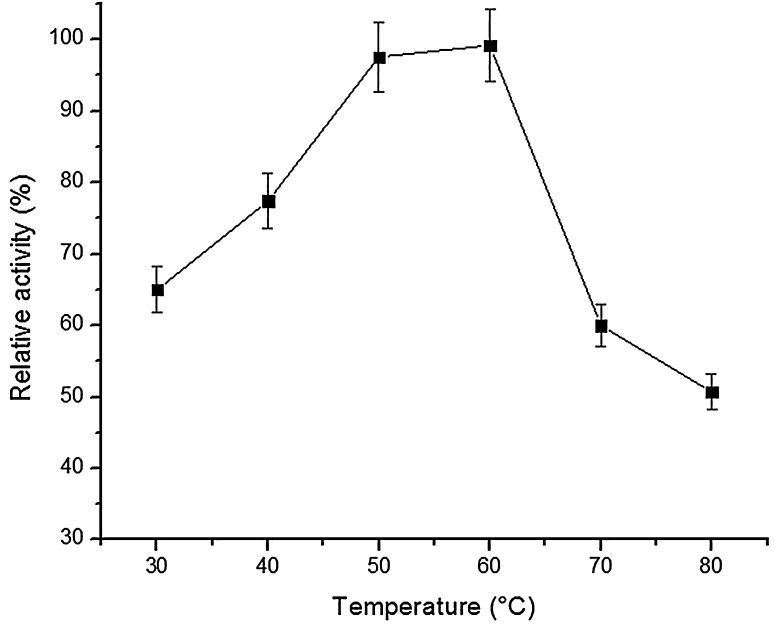
Relative activity of purified lipase from *B. licheniformis* at different temperature ranges

**Fig. 4:**
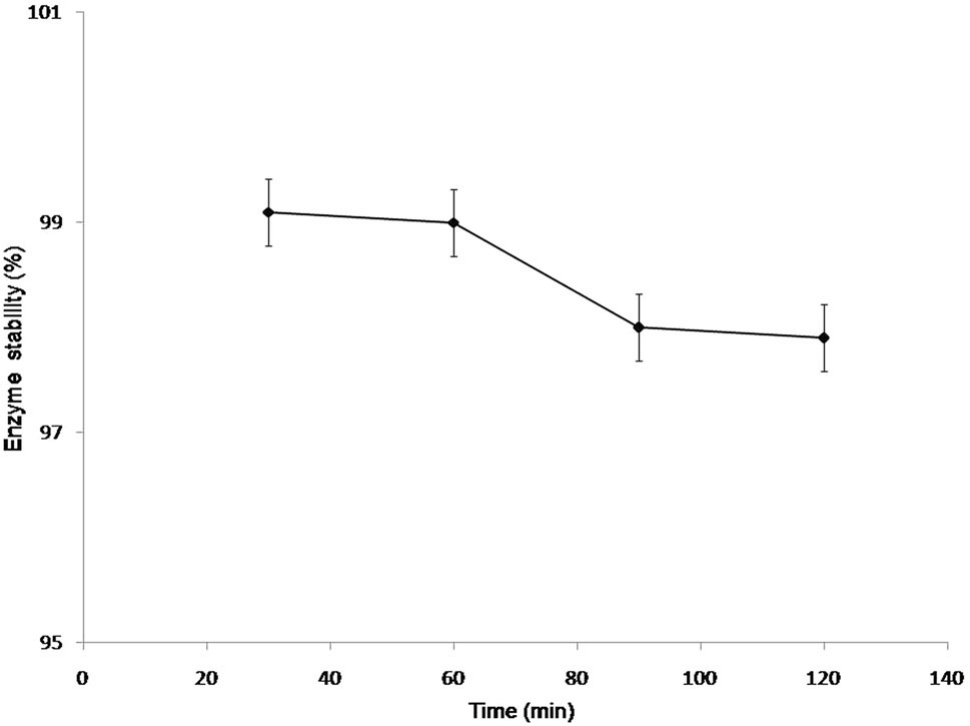
Thermostability profile of purified lipase from *B. licheniformis* at 60 °C

**Fig. 5:**
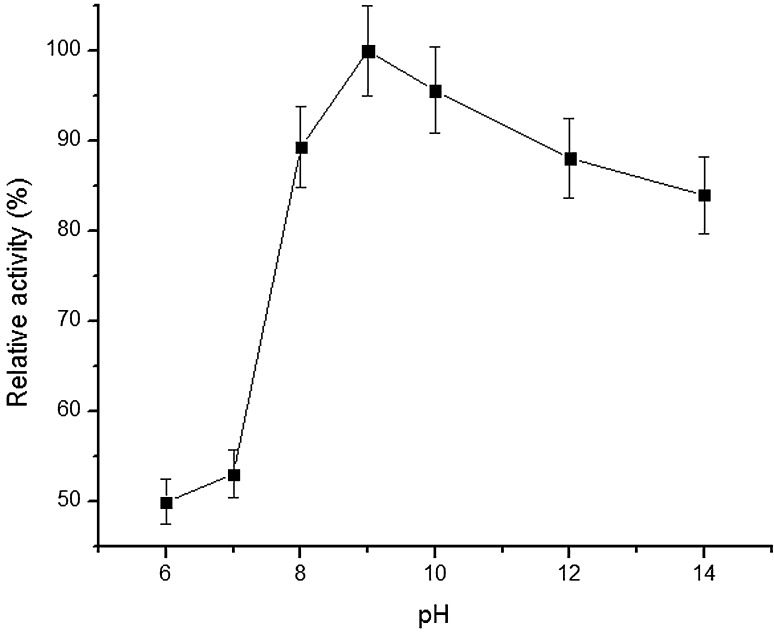
Relative activity of purified lipase from *B. licheniformis* at different pH ranges

The purified lipase from *Bacillus licheniformis* demonstrated optimum temperature activity at 60 °C (Fig. 3), and retained ~99 and 98 % of its original activity when assayed at intervals of 20,40,60,80 and 120 min at 60 °C (Fig. 4). Next, lipase retained activity at wide pH range (6.0–14.0) and was observed to be stable for 1 h under alkaline pH conditions. However, the enzyme activity was considerably at pH 6.0, ~50 % activity was retained after 1 h (Fig. 5).

#### Effect of organic solvents and metal ion on enzyme activity

One of the unique properties of the enzymes is their activity and stability in presence of the organic solvents.Lipase under investigation has shown ~100 % activity in presence of isopropanol and methanol. In presence of acetone and toluene ~60–90 % enzyme activity was retained. Next, effect of metal ions on lipase activity demonstrated that in 1 mM concentration each of Ca^2+^, Mg^2+^, Ni^2+^, and Cu^2+^ lipase displayed 81, 71, 56, and 41 % activity, respectively; whereas, at 10 mM concentration, Mg^2+^ had shown 90 %, Ca^2+^ displayed 85 %, while both Ni^2+^ and Cu^2+^ showed 70 and 63 % enzyme activity.

#### Effect of inhibitors and denaturants on lipase activity

Enzyme activity in presence of inhibitors, β-mercaptoethanol (β-ME), phenylmethylsulfonyl fluoride (PMSF) and diethylpyrocarbonate (DEPC) was tested. At 1 mM concentration both DEPC and PMSF resulted in 98.9 and 86.4 % inhibition, respectively. In contrary, β-ME, an enzyme denaturant has shown 91.8 % inhibition. At 5 mM and 10 mM concentration, the inhibition shown by DEPC, PMSF, β-ME was 94.9, 91, 88 and 93 %, 93 %, 90 %, respectively. PMSF almost completely inhibited enzyme activity at 20 mM concentration, while β-ME and DEPC showed 55.1 and 88.1 % inhibition. Enzyme activity tested in presence of detergent and denaturants revealed that it was able to maintain enzyme activity at 1 and 10 % of Triton X-100 and Tween-20, respectively. Lipase activity was increased from 65.3 to 71.5 % and 63.8 to 67.7 % when treated with Tween-40 and SDS, respectively.

### *In silico* analysis

**Fig. 6:**
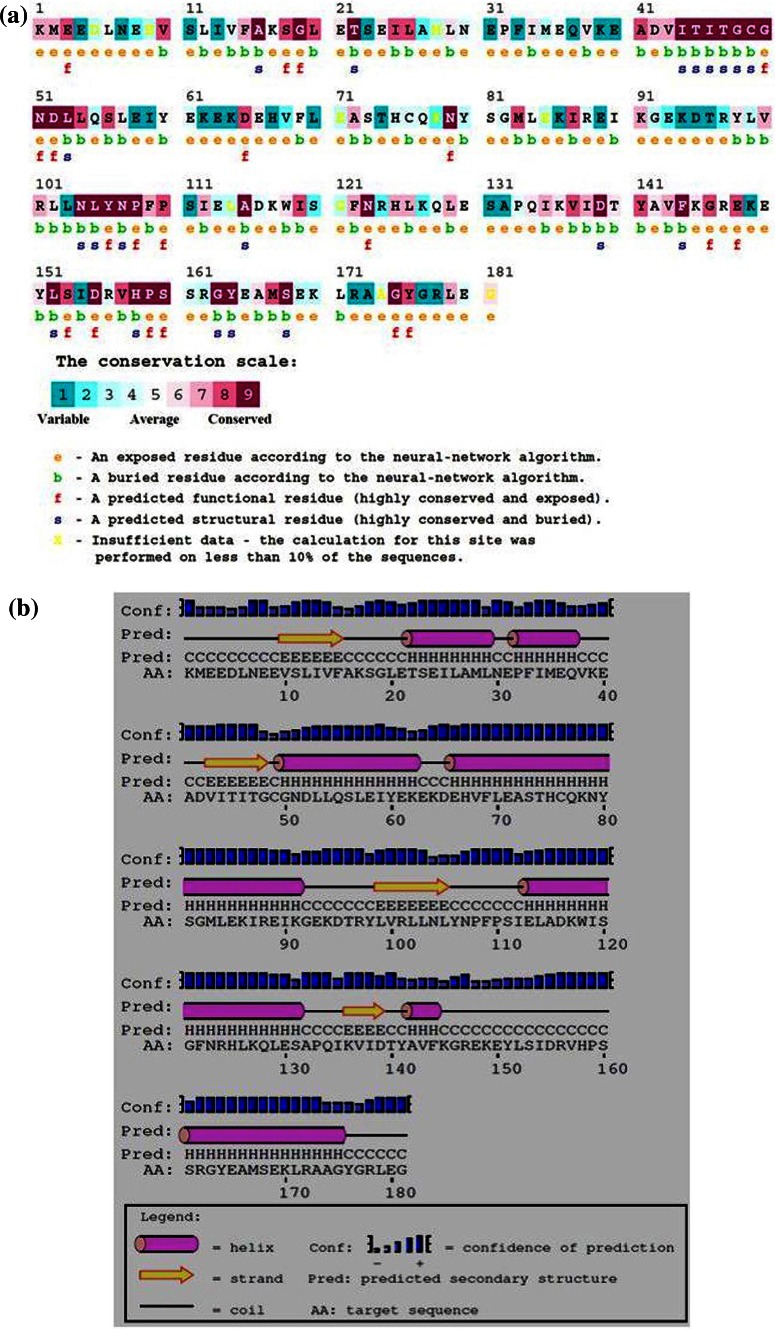
**a** Functionally and structurally important residues identified using ConSeq server, **b** predicted secondary structure using the PSIPRED server

**Fig. 7:**
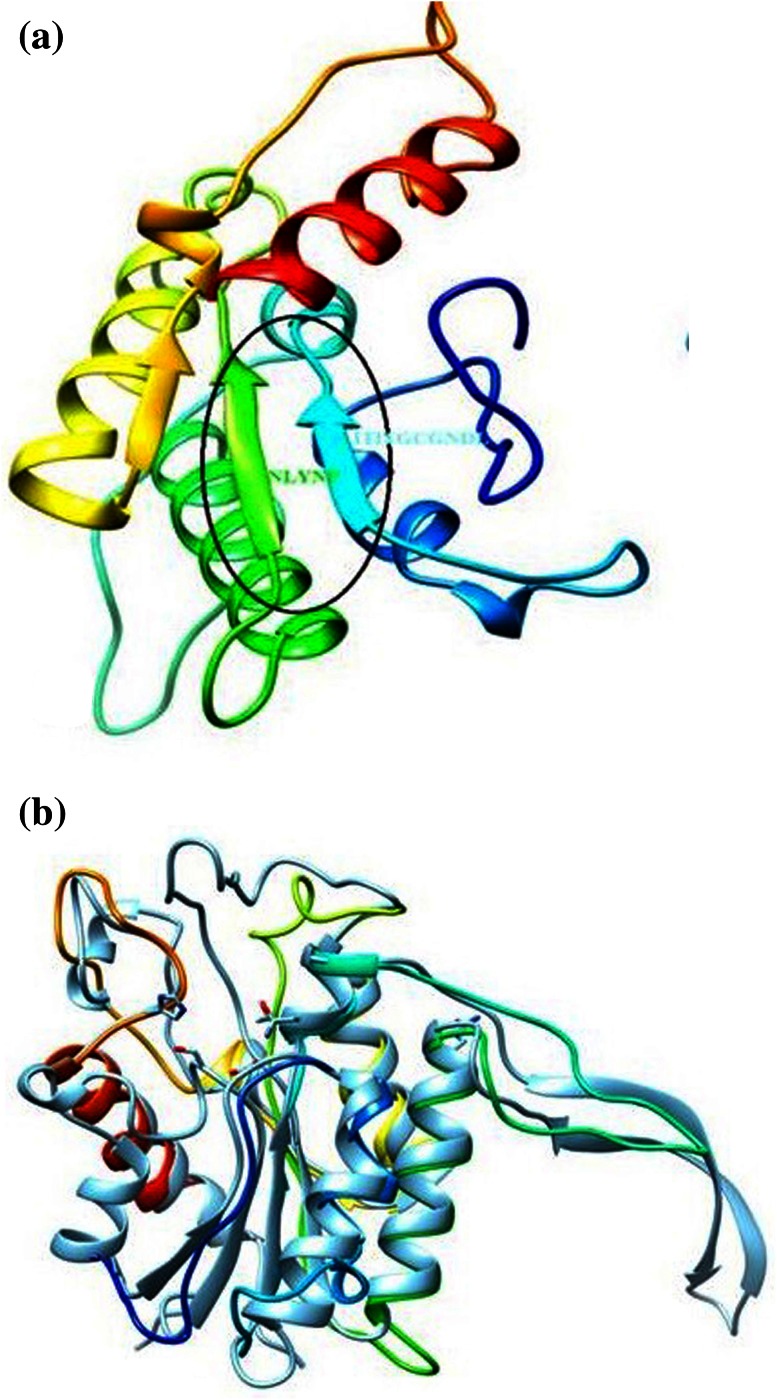
3D comparative model structure: **a** the overall 3D structure and highly conversed region, **b** superimposed structure of model and template structure

Sequence and structural analysis of the lipase gene revealed two highly conserved sequences of ITITGCGNDL and NLYNP, the structurally and functionally important residues were also depicted (Fig. 6a). The secondary structure predicted showed coils helixes and sheets in the structure (Fig. 6b). In addition to this, the 3D model predicted demonstrated a partial α–β hydrolase fold, which has been observed in most of the lipases, interestingly highly conserved sequences observed during MSA were observed to be arranged in a parallel β-sheet at the core of protein. Over all superimposition of the target lipase onto the template showed a scaffold having a core structure and disorder structure in the loop region (Fig. 7a, b).

## Discussion

Recombinant DNA technology has brought great revolution in the field of enzyme industries (Schmidt-Dannert 1999). With the advent of this technology, a number of industrially important enzymes have been cloned and characterized from thermophilic organisms. Interestingly, the thermostable enzymes are preferred more in industrial processes, as most of the industrial processes are carried out at temperatures above 50 °C (Sharma et al. 2002). Higher temperatures offer superior conversion rate, annihilate microbial contamination, increase substrate solubility, help in reducing the viscosity of the reaction medium, favoring mass transfer (Guncheva and Zhiryakova 2011). The present work report cloning, expression, purification and characterization of thermostable lipase from *B. licheniformis* isolated from hot spring soil of Manikaran area of Himachal Pradesh. The mature lipase has 211 amino acids out of which thirty amino acids were coding a terminal signal sequence, as determined by Signal P 4.1 server. This terminal signal sequence helps in secretion of mature lipase out of the cells (Carlsson et al. 2006). Based on the sequence analysis, the lipase gene showed high similarities (~99 %) with most of the *Bacillus* *licheniformis* lipase. Interestingly, the BlastX analysis revealed that the mature lipase has variation in its amino acid sequence, having replacement of a positively charged amino acid lysine (K) to glutamic acid (a negatively charge amino acid). High level expression of the recombinant protein was achieved by inducing T7 promoter by addition of 0.005M IPTG (pET system). The recombinant lipase purified to homogeneity employing Ni–NTA affinity chromatography demonstrated single band of ~22 kDa on SDS-PAGE. The molecular weight of *B. licheniformis* lipase reported from other studies was also observed in the range of 19–25 kDa (Mishra and Madan 2009; Nthangeni et al. 2001; Sharma and Kanwar 2012). Low molecular weight lipases exhibit smaller changes in their tertiary structure during the harsh processes carried out in industries, and hence preferred more (Gray 1993). The low yield of lipases in the present investigation may be attributed to its aggregation behavior, and was also well documented previously (Kambourova et al. 2003; Lesuisse et al. 1993).

Interestingly, the lipase was observed to be quite thermostable and displayed optimum activity and high stability at 60 °C, higher than previously reported lipases from thermostable *B. licheniformis*, where the optimum enzyme activity was observed in the range of 40–55 °C (Annamalai et al. 2011; Chakraborty and Raj 2008; Mishra and Madan 2009, Sangeetha et al. 2010, Sharma and Kanwar 2012). Interestingly, the temperature optimum of **Lip1** displayed comparable activity to the lipase reported by Nthangeni et al. (2001). In addition, the pH optima displayed by the lipase was comparable to the earlier reported studies on *B. licheniformis* (Annamalai et al. 2011; Chakraborty and Raj 2008; Mishra and Madan 2009; Sangeetha et al. 2010; Sharma and Kanwar 2012). The most interesting finding about the pH study is retention of enzyme activity till pH 14, indicating its potential application in detergent-making industry, such observation may be attributed to the ionization of certain amino acids in polypeptide that may favor activity and stability of enzyme at these pH values (Fullbrook 1996), whereas the stability of enzyme at higher temperature is correlated to number of hydrophobic interactions (Goldsack 1970). One unique property of the lipases for which it is explored most is its activity in presence of organic solvent, as enzymes in non-aqueous systems are reported to possess several advantages over the aqueous environment (Klibanov 2001; Sharma and Kanwar 2014). The lipase Lip P1 demonstrated ~100 % activity in presence of the isopropanol and methanol, whereas insignificant inhibition was observed in presence of acetone and toluene. On the contrary, lipase from one of the previous study reported less activity in isopropanol, methanol and acetone, respectively (Chakraborty and Raj 2008); however, the solvent stable features shown by current enzyme was in line with one of the study carried out previously (Rahman et al. 2005). The solvent stability of the lipase may be accredited to retention of the native conformation of the enzyme by forming a protective sheath of water molecules along the hydrophilic surface of enzyme (Nawani and Kaur 2000) that may shield it against the denaturation processes, while increasing the structural flexibility and conformational mobility for optimal catalysis (Klibanov 2001).

Additionally, the metal ion presence in the reaction mix revealed mix effect, Cu^2+^ and Ni^2+^ were relatively showed more inhibition compared to Ca^2+^ and Mg^2+^. The decrease in the enzyme activity suggests that metal ion might be competing at the active site of the enzyme, as also documented in previous studies (Ghori et al. 2011). Previous studies have also shown miscellaneous effect of metal ions on enzyme activity (Annamalai et al. 2011; Chakraborty and Raj 2008). Transition metals (Cu^2+^ and Ni^2+^) reported to hinder the enzyme activity via interacting with side chain groups of surface amino acids that may affect the conformational stability of enzyme (Rahman et al. 2006). Inhibition by Cu^2+^ evinces the presence of sulphydryl group in the protein (Enujiugha et al. 2004; Sanders and Pattee 1975). Numerous studies have also talked about the inhibition of lipase activity in presence of Ni^2+^ and Cu^2+^ (Kumar et al. 2005; Ma et al. 2006). In contrast to this, the higher activity of lipase in presence of Ca^2+^ may be ascribed to the formation of long chain insoluble fatty acid calcium salts during hydrolysis, plus formation of bridges at the active site that help in improving the enzyme stability (Kim et al. 2000; Wills 1960). Further analysis of the effect of the enzyme inhibitors on enzyme activity demonstrated that both PMSF and DEPC, resulted in inhibition of the enzyme activity that principally indicates presence of serine and histidine in the active center. The catalytic center is suggested to be consisting of a catalytic triad with Ser–His–Asp/Glu (Gupta et al. 2004; Shariff et al. 2011).

Inhibition by PMSF and DEPC illustrates easy accessibility of inhibitors to the active site serine and histidine that may attribute to lid structure absence in the present lipase (lid structure covers the catalytic site) (Lesuisse et al. 1993). The β-ME also inhibited the enzyme activity to the marked level, which further ruled out the possibility of disulfide linkage in the protein structure (Abramic et al. 1999). Similar inhibitory effects of PMSF, DEPC and β-ME on lipase activity were also reported from earlier studies (Castro-Ochoa et al. 2005; Chakraborty and Raj 2008; Lee et al. 1999; Mishra and Madan 2009; Nthangeni et al. 2001; Shariff et al. 2011). In sharp contrast to this, 100 % lipase activity of *B. licheniformis* VSG1 in presence of PMSF was reported by Sangeetha et al. (2010); in many studies, β-ME was also not reported to inhibit the lipase activity (Castro-Ochoa et al. 2005; Kanjanavas et al. 2010; Lee et al. 1999; Sharma et al. 2012).

More interestingly, effect of denaturants/detergents on lipase activity were quite dissimilar from other studies, as Triton X-100 and Tween 20 were observed to be inhibitory, and Tween 40 and SDS showed enhanced enzyme activity, as concentration of the chemical modifiers was raised from 1 to 10 %. Previous studies have reported 100 % lipase activity in presence of Triton X-100 (Annamalai et al. 2011; Balan et al. 2013; Castro-Ochoa et al. 2005; Sangeetha et al. 2010; Sharma et al. 2002), Mishra and Madan (2009) reported 90 % lipase activity, whereas Kanjanavas et al. (2010) reported 41.6 % lipase activity. More interestingly, the activity in presence of SDS was higher in contrast to previous studies carried out with *Bacillus* sp. (Annamalai et al. 2011; Castro-Ochoa et al. 2005; Lee et al. 1999; Mishra and Madan 2009). These observations make this enzyme fit for the detergent-making industries. The effect of Tween 20 on lipase activity was observed to be analogous as reported in other studies (Kanjanavas et al. 2010; Mishra and Madan 2009; Sharma et al. 2002). This may be attributed to the ability of the detergents to hamper enzyme aggregation by weakening hydrophobic interaction and improving the substrate accessibility to the enzyme (Polizelli et al. 2008).

The predicted secondary structure of the lipase contains helix, coil and sheet. Interestingly, important residues predicted through ConSeq server showed number of amino acids that were exposed and buried, and further divulge that the residues present towards the N terminus and C terminus of the protein were highly exposed, and might be interacting with the aqueous environment to form network of hydrogen bonds that help in stabilizing the 3D structure; *in silico* analysis of the lipase revealed no significant similarity with the previously reported X-ray crystal structure. However, crystal structure of a putative lipase from *Bacteroides thetaiotaomicron* (PDB ID: 3BZW) showed ~20 % similarity with the lipase under investigation and directed us to predict the 3D structure. Two highly conserved sequences were observed in the protein that contains amino acid sequences consisting of ITITGCGNDL and NLYNP, respectively, which were arranged as parallel β-sheet in the core structure, further analysis of the conserved sequences revealed a defined secondary structure, with loop region observed to be disordered. Such observation may be attributed to the presence of amino acids which confer structural flexibility. Another reason could be the incomplete prediction of 3D structure on a template that had shared only 20 % structural similarity, nonetheless, the predicted structure can be explored in future to under- stand the structure and function of this lipase.

## Conclusion

In this investigation, a lipase produced by *B. licheniformis* was cloned, overexpressed and purified. Of particular note, the lipase was observed to be active at wide range of temperature and pH and displayed activity in presence of organic solvent, metal ions and detergents. By virtue of all these properties, this thermo-alkali-tolerant lipase can be explored for its industrial applications. Next, we aimed at improving the structure and functional stability of the lipase by site-directed mutagenesis.
